# Retraction Note: Synthesis, antimicrobial, anti-inflammatory, antioxidant and cytotoxicity of new pyrimidine and pyrimidopyrimidine derivatives

**DOI:** 10.1038/s41598-026-65328-4

**Published:** 2026-08-04

**Authors:** Reda Mohammed Keshk, Zeinab Ahmed Salama, Samir Kamel Elsaedany, Elsayed Mahmoud Abed ElRehim, Doha Mohammad Beltagy

**Affiliations:** 1 Chemistry Department, Faculty of Science, Damanhour University, Damanhour, 22511 Egypt; 2 Chemistry Department, Faculty of Science, Alexandaria University, Alexandaria, Egypt; 3 Biochemistry Department, Faculty of Science, Damanhour University, Damanhour, 22511 Egypt

Retraction of: *Scientific Reports* 10.1038/s41598-025-92066-w, published online 18 March 2025

The Editors have retracted this Article.

Following publication, concerns were raised regarding the reliability of the analytical data, including inconsistencies in the mass spectrometry results (molecular ion assignment and chlorine isotope pattern) and physically implausible reported ^1^H–^1^H J-coupling constants. Although the Authors provided revised spectra and explanations, they were unable to supply the original raw NMR and mass spectrometry data for independent verification, and the discrepancies were not satisfactorily resolved. In the absence of verifiable raw data and given the unresolved analytical inconsistencies, the Editors no longer have confidence in the reliability of the results reported in this Article.

Reda Mohammed Keshk disagrees with the retraction. None of the other Authors responded to the correspondence regarding this retraction.

